# Impact of obesity on intensive care unit outcomes in older patients with critical illness: A cohort study

**DOI:** 10.1371/journal.pone.0297635

**Published:** 2024-02-14

**Authors:** Shan Li, Zhiqing Fu, Wei Zhang, Hongbin Liu

**Affiliations:** Department of Cardiology, The Second Medical Center & National Clinical Research Center for Geriatric Diseases, Chinese PLA General Hospital, Beijing, China; National Institutes of Health, UNITED STATES

## Abstract

**Background:**

Although the paradoxical association between obesity and improved survival has been reported in a variety of clinical settings, its applicability to intensive care unit (ICU) outcomes in older critically ill patients remains unclear. We sought to examine the association between obesity and 30-day mortality and other adverse outcomes in this population.

**Methods:**

We analyzed data of older patients (≥ 60 years) in the eICU Collaborative Research Database. Body mass index (BMI) was stratified according to the World Health Organization obesity classification. Logistic regression model was used to estimate adjusted odds ratios (ORs), and cubic spline curve was used to explore the nonlinear association between BMI and 30-day ICU outcomes. Stratified analysis and sensitivity analysis were also performed.

**Results:**

Compared with class I obesity, under- and normal-weight were associated with higher all-cause, cardiovascular and noncardiovascular mortality, and class III obesity was associated with greater all-cause and cardiovascular mortality (OR, 1.18 [95% CI, 1.06–1.32], 1.28 [1.08–1.51]). Obesity classes II and III were associated with higher composite all-cause mortality, mechanical ventilation, or vasoactive drug usage risks (OR, 1.12 [95% CI, 1.04–1.20], 1.33 [1.24–1.43]). Mechanical ventilation was strongly positively associated with BMI. A significant BMI-by-sex interaction was observed for cardiovascular mortality, such that the association between severe obesity and mortality was more pronounced among older men than older women.

**Conclusions:**

The obesity paradox does not appear to apply to short-term ICU outcomes in older patients with critical illness, mainly due to increased all-cause and cardiovascular mortality in severely obese patients, particularly in men.

## Introduction

The prevalence of obesity among U.S. adults has risen significantly, with a reported age-adjusted prevalence of 42.4% in 2017–2018 [1]. As the prevalence of obesity increases worldwide, so does the number of obese patients admitted to the intensive care units (ICUs), accounting for approximately 30% [2,3]. Although obesity is recognized as a contributor to a variety of physiological disorders [4], there is emerging evidence suggesting a counterintuitive association between obesity and improved survival, the so-called obesity-survival paradox [5–8]. Obese individuals admitted to the ICU have a greater burden of comorbidity, have less physiological reserve to cope with the stress of critical illness, and therefore theoretically have a higher risk of death and other adverse outcomes such as respiratory and circulatory failure, the latter of which leads to an increased dependence on mechanical ventilation and vasoactive medications. However, previous studies have shown that obese patients have better survival than their normal weight counterparts in the critical care setting [9,10]. These findings are based primarily on young and middle-aged patients. Ageing-related physiologic changes, including loss of lean body mass and redistribution of adipose tissue, may add uncertainty to the impact of obesity on clinical outcomes in older critically ill patients.

The global population is rapidly ageing, with approximately 21% of the population is expected to reach 60 years or older by 2050 [11]. Accordingly, ICUs are faced with an increasing care demand for older patients. Older patients admitted to the ICU are frail, disabled, and more likely to have treatment intolerance and insensitivity than young and middle-aged patients. Obesity-related proinflammatory, prothrombotic, insulin resistance and vasoconstrictive states are potential predisposing factors for adverse consequences [12]. It is currently unclear how the collision of the two major prognostic factors, ageing and obesity, will affect the clinical outcomes of critically ill patients. In particular, whether the protective effect of obesity, known as the obesity paradox, remains evident in older critically ill patients. To address this evidence gap, we investigated the association between body mass index (BMI) and 30-day ICU outcomes in older critically ill patients using a large ICU database. The aim of this study was threefold: first, to assess the relationship between BMI and all-cause and cause-specific mortality; second, to examine the effect of BMI on other adverse events (mechanical ventilation and vasoactive drug usage); and third, to determine whether sex- or age-related differences therein.

## Methods

### Data sources and participants

All data are available through the publicly accessible eICU Collaborative Research Database Platform (https://www.physionet.org). The eICU database is a large-scale telemedicine system developed by Philips Healthcare and Massachusetts Institute of Technology to support and improve the management of critically ill patients in ICUs. A method of stratified random sampling was employed to select patients for inclusion in the public database. It is a multicenter database with a high level of data granularity, covering more than 200,000 admissions from 2014 to 2015 in 335 ICUs at 208 hospitals in the United States [13]. The database contains a large amount of demographic information, physiologic parameters, physical examination, severity assessment, admission diagnoses, medical history, laboratory measurements, fluid loads, medications, and care plan information. Data are available after completing a training course in human subjects research and signing a data usage agreement. One author (Shan Li) obtained database access and was responsible for data extraction (certification number: 46622368). The requirement for patient consent and an ethical approval statement was waived by the Massachusetts Institute of Technology due to the retrospective design, lack of patient intervention and de-identification of all patient information. This study was conducted in accordance with the principles of the Declaration of Helsinki. The study followed the Strengthening the Reporting of Observational Studies in Epidemiology (STROBE) guidelines (S1 Checklist). The study population included older patients (≥ 60 years) admitted to the ICU. After excluding patients without available weight or height measurements and those with extreme BMIs of less than 10 kg/m^2^ or greater than 60 kg/m^2^, the final analysis included 89234 individuals.

### Exposure

The primary exposure was BMI, calculated by the equation BMI (kg/m^2^) = weight/height^2^. The weight and height recorded at ICU admission were used for this calculation. BMI was stratified by World Health Organization (WHO) obesity classification, defined as underweight <18.5 kg/m^2^, normal weight 18.5–24.9 kg/m^2^, overweight 25.0–29.9 kg/m^2^, class I obesity (mild obesity) 30.0–34.9 kg/m^2^, class II obesity (moderate obesity) 35.0–39.9 kg/m^2^, and class III obesity (severe obesity) ≥ 40.0 kg/m^2^. To facilitate the interpretation of the results, we defined the group with the lowest risk as the reference group, which is class I obesity.

### Outcomes

The primary outcomes were all-cause mortality and cause-specific mortality (including cardiovascular and noncardiovascular mortality) within 30 days of ICU admission. The secondary outcomes included major adverse events (a composite of all-cause mortality, mechanical ventilation, or vasoactive drug usage), mechanical ventilation, and vasoactive drug usage. All-cause death was defined as death from any cause. Cardiovascular death was defined as death from the International Classification of Disease, 9th Revision, codes 390–459.

### Covariates

The following covariates were included in the analysis: age, sex, ethnicity, mean blood pressure and heart rate, disease severity score (Glasgow coma score [GCS] and Acute Physiology, Age and Chronic Health Evaluation [APACHE] score), primary admission disease (circulatory disease, respiratory disease, neurological disease, digestive disease, genitourinary disease, trauma, and other diseases), prior comorbidities (coronary artery disease, stroke/transient ischaemic attacks, diabetes mellitus, hypertension, chronic heart failure, chronic obstructive pulmonary disease, dementia, cirrhosis, peripheral artery disease, renal dysfunction), and important treatments (mechanical ventilation, dialysis, vasoactive drugs). The database contains the APACHE score and GCS data, which are estimated based on the worst values of physiologic parameters and laboratory measurements during the first 24 hours of ICU admission. The diagnoses of primary admission disease were defined according to ICD-9 codes. In addition, we considered other patient-level and hospital-level factors (admission source, ICU bed count, geographic location, and year of discharge) in the sensitivity analysis.

### Statistical analysis

Statistical analyses were performed with R software (version 3.6.1, http://www.r-project.org) and EmpowerStats (http://www.empowerstats.com, X&Y Solutions, Inc., Boston, MA). Two-sided P values of < 0.05 indicated statistical significance.

Summary statistics for continuous variables are presented as either mean (standard deviation) or median (interquartile range), while categorical variables are shown as counts (proportions). Differences in baseline characteristics between BMI categories were compared by ANOVA, Kruskal‒Wallis test, or chi-squared tests. We used multiple imputation, based on 5 replications and a chained equation approach in the R MI procedure, to account for missing covariate data. The percentage of missing data was as follows: 13.2% for APACHE score, 2.3% for GCS, 1.0% for mean blood pressure, and 0.8% for heart rate. The logistic regression model was used to examine the association between categorical BMI and the primary and secondary outcomes. We constructed three models: model I, unadjusted, model II, adjusted for age, sex and ethnicity, and model III, adjusted for all predefined covariates. Models were not adjusted for mechanical ventilation or vasoactive drug usage as covariates when they were treated as outcomes. The cubic spline curve based on the generalized additive model was used to explore the nonlinear association between continuous BMI and the primary and secondary outcomes, with adjusted for all predefined covariates. To account for the effect modification of sex or age, the multiplicative interaction term was tested in the multivariate model. Several sensitivity analyses were performed to verify the robustness of results. (1) An analysis was performed by excluding those who died within 24 hours of ICU admission to mitigate the reverse causality. (2) A model was constructed in patients without a history of cancer to rule out the influence of cancer on the association between BMI and survival. (3) An analysis was also conducted that excluded patients with diabetes to eliminate the effect of diabetes on obesity-related clinical outcomes. (4) A complete case analysis was performed by excluding all missing covariate data to check whether the missing data modified the current findings. (5) A Cox proportional hazards model analysis was performed using ICU length of stay as a potential time scale to account for possible differences in outcomes between statistical methods. (6) Other confounding factors were further addressed, such as admission source, hospital location, ICU bed count, and discharge year, and we constructed an additional model adjusting for these variables in addition to all the other variables.

## Results

### Study participants

The baseline characteristics of patients in different BMI categories are shown in Table 1. The study cohort had a median age of 74.1 (SD 8.9) years with slight male predominance (53.3%) and was ethnically diverse, with Caucasians in the majority (80.7%). The average BMI was 28.3 (SD 7.2) kg/m^2^. Individuals with a higher BMI were younger and more likely to have a history of diabetes mellitus, hypertension, heart failure and renal dysfunction. More dependence on mechanical ventilation was observed among patients with class III obesity. Underweight and class III obese individuals accounted for 4.5% and 6.9% of the total population, resulting in all-cause mortality rates of 17.1% and 10.2%, while class I obese individuals accounted for 18.5% of the total population, resulting in an all-cause mortality rate of 9.0%. The distribution of BMI categories in the total population and in men and women is shown in S1 Fig.

**Table 1:** Baseline characteristics of individuals by BMI categories.

BMI, kg/m^2^	<18.5	18.5–24.9	25.0–29.9	30.0–34.9	35.0–39.9	≥40	P value
N (%)	4016 (4.5)	27286 (30.6)	27616 (30.9)	16543 (18.5)	7632 (8.6)	6141 (6.9)	
Age, years	76.5 ±10.2	76.5 ±10.0	74.6 ±9.2	72.8 ±8.5	71.4 ±7.9	69.7 ±7.1	<0.001
Male, n (%)	1576 (39.2)	14301 (52.4)	16380 (59.3)	9172 (55.4)	3715 (48.7)	2397 (39.0)	<0.001
Ethnicity							
Caucasian, n (%)	3153 (78.5)	21676 (79.4)	22276 (80.7)	13595 (82.2)	6273 (82.2)	5045 (82.2)	<0.001
African American, n (%)	405 (10.1)	2238 (8.2)	2130 (7.7)	1319 (8.0)	709 (9.3)	642 (10.5)	<0.001
Hispanic, n (%)	118 (2.9)	1046 (3.8)	1115 (4.0)	553 (3.3)	227 (3.0)	158 (2.6)	<0.001
Other/unknown, n (%)	340 (8.5)	2326 (8.5)	2095 (7.5)	1076 (6.6)	423 (5.5%)	296 (4.8)	<0.001
BMI, kg/m^2^	16.7 ±1.5	22.3 ±1.8	27.3 ±1.4	32.2 ±1.4	37.1 ±1.4	45.5 ±4.9	<0.001
Heart rate, bpm	103 ±32	98 ± 33	95 ±33	94 ±32	95 ±32	95 ±33	<0.001
Mean arterial pressure, mmHg	81 ±41	83 ± 41	85 ±42	86 ±43	88 ±44	89 ±44	<0.001
Severity score							
APACHE score	57 (41–75)	54 (40–71)	52 (37–68)	51 (37–68)	52 (38–68)	53 (38–70)	<0.001
GCS	12 ±4	13 ±4	13 ±4	13 ±4	13 ±4	13 ±4	<0.001
Primary reason of ICU admission							
Cardiovascular disease, n (%)	1213 (30.2)	10986 (40.3)	13114 (47.5)	8066 (48.8)	3481 (45.6)	2430 (39.6)	<0.001
Respiratory disease, n (%)	1220 (30.4)	5378 (19.7)	4297 (15.6)	2568 (15.5)	1368 (17.9)	1351 (22.0)	<0.001
Digestive disease, n (%)	487 (12.1)	3453 (12.7)	3100 (11.2)	1628 (9.8)	728 (9.5)	572 (9.3)	<0.001
Genitourinary disease, n (%)	228 (5.7)	1589 (5.8)	1394 (5.0)	916 (5.5)	464 (6.1)	469 (7.6)	<0.001
Neurological disease, n (%)	237 (5.9)	1345 (4.9)	1316 (4.8)	767 (4.6)	360 (4.7)	272 (4.4)	0.012
Trauma, n (%)	126 (3.1)	1095 (4.0)	916 (3.3)	474 (2.9)	168 (2.2)	102 (1.7)	<0.001
Other infectious disease, n (%)	143 (3.6)	923 (3.4)	943 (3.4)	612 (3.7)	327 (4.3)	328 (5.3)	<0.001
Other disease, n (%)	362 (9.0)	2517 (9.2)	2536 (9.2)	1512 (9.1)	736 (9.6)	617 (10.0)	<0.001
Pre-admission comorbidities							
Coronary artery disease, n (%)	719 (17.9)	6208 (22.8)	7109 (25.7)	4276 (25.8)	1907 (25.0)	1342 (21.9)	<0.001
Stroke/TIA, n (%)	504 (12.5)	3569 (13.1)	3458 (12.5)	1918 (11.6)	779 (10.2)	598 (9.7)	<0.001
Diabetes mellitus, n (%)	240 (6.0)	2074 (7.6)	3018 (10.9)	2560 (15.5)	1539 (20.2)	1615 (26.3)	<0.001
Hypertension, n (%)	1791 (44.6)	13964 (51.2)	15084 (54.6)	9448 (57.1)	4416 (57.9)	3548 (57.8)	<0.001
Congestive heart failure, n (%)	560 (13.9)	4321 (15.8)	4486 (16.2)	2943 (17.8)	1586 (20.8)	1633 (26.6)	<0.001
Peripheral arterial disease, n (%)	209 (5.2)	1568 (5.7)	1625 (5.9)	911 (5.5)	426 (5.6)	307 (5.0)	0.065
Chronic obstructive pulmonary disease, n (%)	1059 (26.4)	4638 (17.0)	3896 (14.1)	2423 (14.6)	1319 (17.3)	1366 (22.2)	<0.001
Renal insufficiency, n (%)	428 (10.7)	3419 (12.5)	3631 (13.1)	2261 (13.7)	1099 (14.4)	1046 (17.0)	<0.001
Dementia	337 (8.4)	1744 (6.4)	1183 (4.3)	499 (3.0)	181 (2.4)	115 (1.9)	<0.001
Cirrhosis	81 (2.0)	601 (2.2)	552 (2.0)	331 (2.0)	151 (2.0)	125 (2.0)	<0.001
Theraputics							
Mechanical ventilation, n (%)	997 (24.8)	6181 (22.7)	6321 (22.9)	4124 (24.9)	2173 (28.5)	2076 (33.8)	<0.001
Dialysis, n (%)	148 (3.7)	1028 (3.8)	955 (3.5)	554 (3.3)	270 (3.5)	211 (3.4)	0.231
Vasoactive drugs, n (%)	174 (4.3)	1140 (4.2)	1224 (4.4)	672 (4.1)	330 (4.3)	244 (4.0)	0.374
Admission source							
Emergency department, n (%)	2289 (57.0)	14255 (52.2)	13517 (48.9)	7674 (46.4)	3657 (47.9)	3089 (50.3)	<0.001
Acute care/floor, n (%)	723 (18.0)	4375 (16.0)	4028 (14.6)	2467 (14.9)	1167 (15.3)	1111 (18.1)	<0.001
Other, n (%)	1004 (25.0)	8656 (31.7)	10071 (36.5)	6402 (38.7)	2808 (36.8)	1941 (31.6)	<0.001
Geographic location							
Midwest, n (%)	1164 (29.0)	8382 (30.7)	9156 (33.2)	5916 (35.8)	2859 (37.5)	2430 (39.6)	<0.001
South, n (%)	1263 (31.4)	7988 (29.3)	7970 (28.9)	4656 (28.1)	2074 (27.2)	1669 (27.2)	<0.001
West, n (%)	839 (20.9)	5920 (21.7)	5843 (21.2)	3306 (20.0)	1439 (18.9)	1075 (17.5)	<0.001
Northeast, n (%)	270 (6.7)	1779 (6.5)	1812 (6.6)	1175 (7.1)	587 (7.7)	501 (8.2)	<0.001
Unknown, n (%)	480 (12.0)	3217 (11.8)	2835 (10.3)	1490 (9.0)	673 (8.8)	466 (7.6)	<0.001
Bedcount							
≤30	336 (8.4)	1960 (7.2)	1980 (7.2)	1176 (7.1)	549 (7.2)	487 (7.9)	<0.001
31–60	2667 (66.4)	18071 (66.2)	18146 (65.7)	10798 (65.3)	4948 (64.8)	3982 (64.8)	<0.001
61–90	644 (16.0)	4820 (17.7)	5061 (18.3)	3107 (18.8)	1451 (19.0)	1100 (17.9)	<0.001
91–120	369 (9.2)	2435 (8.9)	2429 (8.8)	1462 (8.8)	684 (9.0)	572 (9.3)	<0.001
Year of discharge							0.659
2014, n (%)	1913 (47.6)	12871 (47.2)	12870 (46.6)	7818 (47.3)	3573 (46.8)	2889 (47.0)	
2015, n (%)	2103 (52.4)	14415 (52.8)	14746 (53.4)	8725 (52.7)	4059 (53.2)	3252 (53.0)	
ICU length of stay, days	5.2 (2.9–8.6)	5.0 (2.8–8.4)	4.9 (2.7–8.1)	4.9 (2.8–8.2)	5.1 (2.9–8.9)	5.6 (3.1–9.6)	<0.001
All-cause death, n (%)	685 (17.1)	3375 (12.4)	2715 (9.8)	1488 (9.0)	731 (9.6)	629 (10.2)	<0.001
Cardiovascular death, n (%)	216 (5.4)	1180 (4.3)	1158 (4.2)	650 (3.9)	321 (4.2)	261 (4.3)	<0.001
Noncardiovascular death, n (%)	469 (11.7)	2195 (8.0)	1557 (5.6)	838 (5.1)	410 (5.4)	368 (6.0)	<0.001

Values are mean (standard deviation), median (inter-quartile range) or number (percentage). APACHE, acute physiology, age and chronic health evaluation. GCS, glasgow coma score.

### The primary outcomes

**Fig 1:**
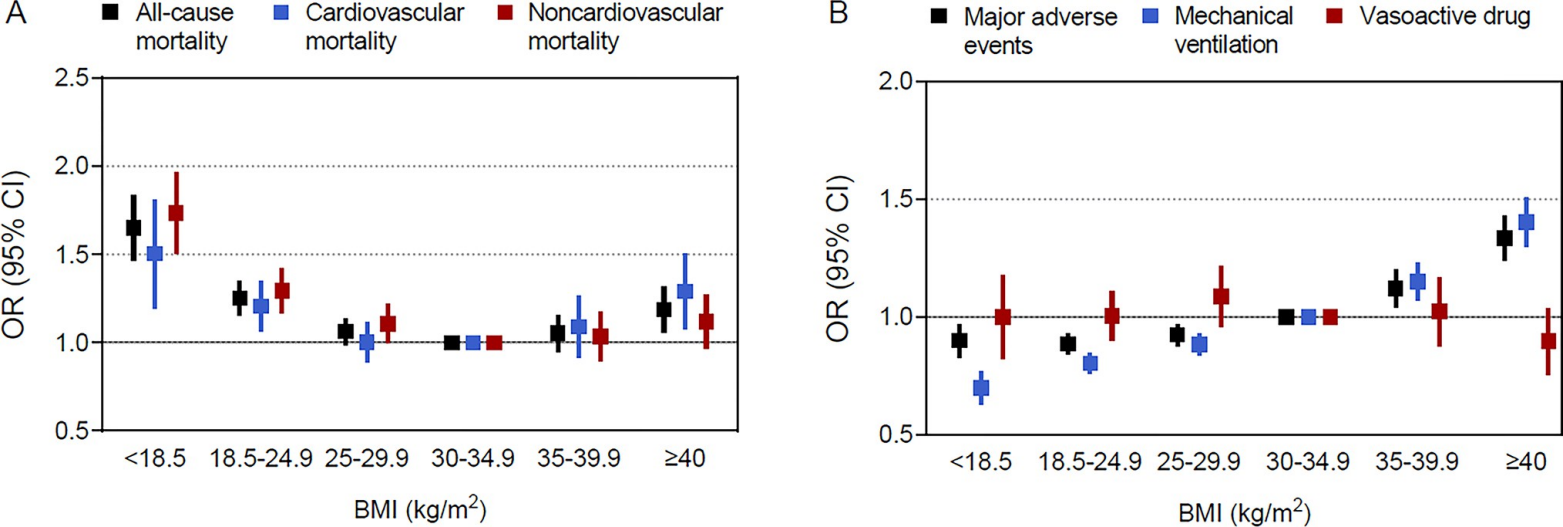
Multivariable adjusted odds ratios for primary and secondary outcomes by categorical BMI. (A) all-cause mortality, cardiovascular mortality, and noncardiovascular mortality. (B) major adverse events (composite of all-cause mortality, mechanical ventilation, and vasoactive drugs), mechanical ventilation, and vasoactive drug. Outcomes were adjusted for all predefined covariates.

**Fig 2:**
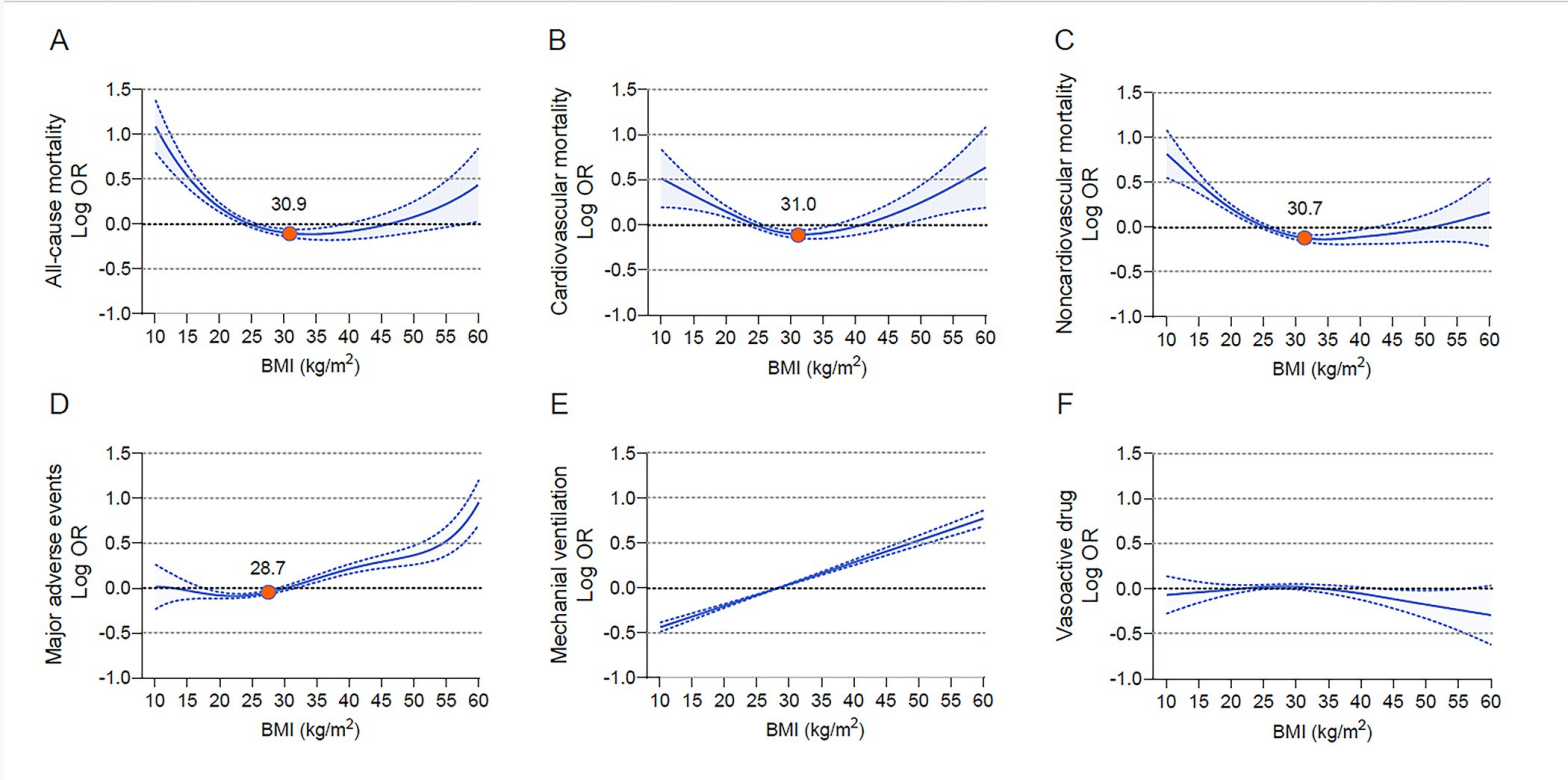
Multivariable adjusted odds ratios for primary and secondary outcomes by continuous BMI. (A) All-cause mortality. (B) cardiovascular mortality. (C) noncardiovascular mortality. (D) major adverse events (composite of all-cause mortality, mechanical ventilation, and vasoactive drugs). (E) mechanical ventilation. (F) vasoactive drug. Outcomes were adjusted for all predefined covariates.

All-cause death occurred in 9623 (10.8%) and cardiovascular death occurred in 3786 (4.2%) patients. Compared with class I obesity, under- and normal-weight were associated with higher all-cause, cardiovascular and noncardiovascular mortality. Notably, class III obesity was also associated with greater all-cause and cardiovascular mortality (OR, 1.18 [95% CI, 1.06–1.32], 1.28 [1.08–1.51]), but not with noncardiovascular death (OR, 1.11 [95% CI, 0.97–1.28]) (Fig 1 and S1 Table). Accordingly, there was a U-shaped association between continuous BMI and all-cause and cardiovascular death, whereas a reverse J-shaped association was observed for noncardiovascular death. The lowest risk of all-cause, cardiovascular and noncardiovascular death occurred at BMI inflection points of 30.9, 31.0 and 30.7, respectively (Fig 2). After the inflection points, for every 5 kg/m^2^ increase in BMI, the risk of all-cause and cardiovascular mortality increased by 7% and 11%, while the risk of noncardiovascular mortality did not change significantly (S2 Table).

### The secondary outcomes

Major composite adverse events, mechanical ventilation, and vasoactive drug usage occurred in 28331 (31.8%), 21872 (24.5%), and 3784 (4.2%) patients, respectively. Compared with class I obesity, class II and class III obesity were associated with an increased risk of major composite adverse events, while underweight, normal weight and overweight were associated with a decreased risk. Mechanical ventilation had a strong, progressive linear association with increasing BMI, whereas no significant association of BMI category with vasoactive drug usage was observed (Fig 1 and S3 Table). Correspondingly, a J-shaped association between BMI and major composite adverse events was obvious, with the lowest risk at a BMI inflection point of 28.7. After the inflection point, every 5 kg/m^2^ increase in BMI was associated with a 15% higher risk. The relationship between BMI and mechanical ventilation was monotonically linear, with every 5 kg/m^2^ increase in BMI associated with an 11% higher risk (Fig 2 and S2 Table).

### Sex and age interactions on the association between BMI and outcomes

**Fig 3:**
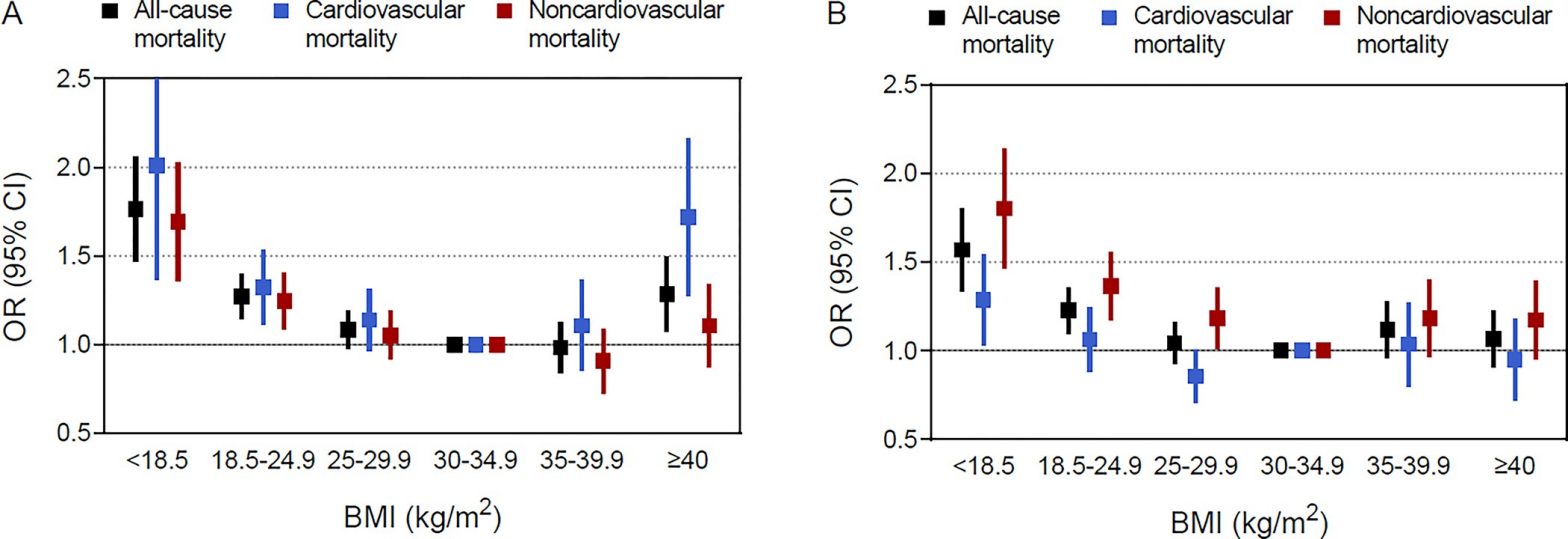
Multivariable adjusted odds ratios for primary outcomes by categorical BMI among men and women. (A) Adjusted odds ratios and 95% confidence intervals for men. (B) adjusted odds ratios and 95% confidence intervals for women. Outcomes were adjusted for all predefined covariates except sex. P for sex interaction: all-cause mortality 0.23, cardiovascular mortality 0.01, and noncardiovascular mortality 0.56.

**Fig 4:**
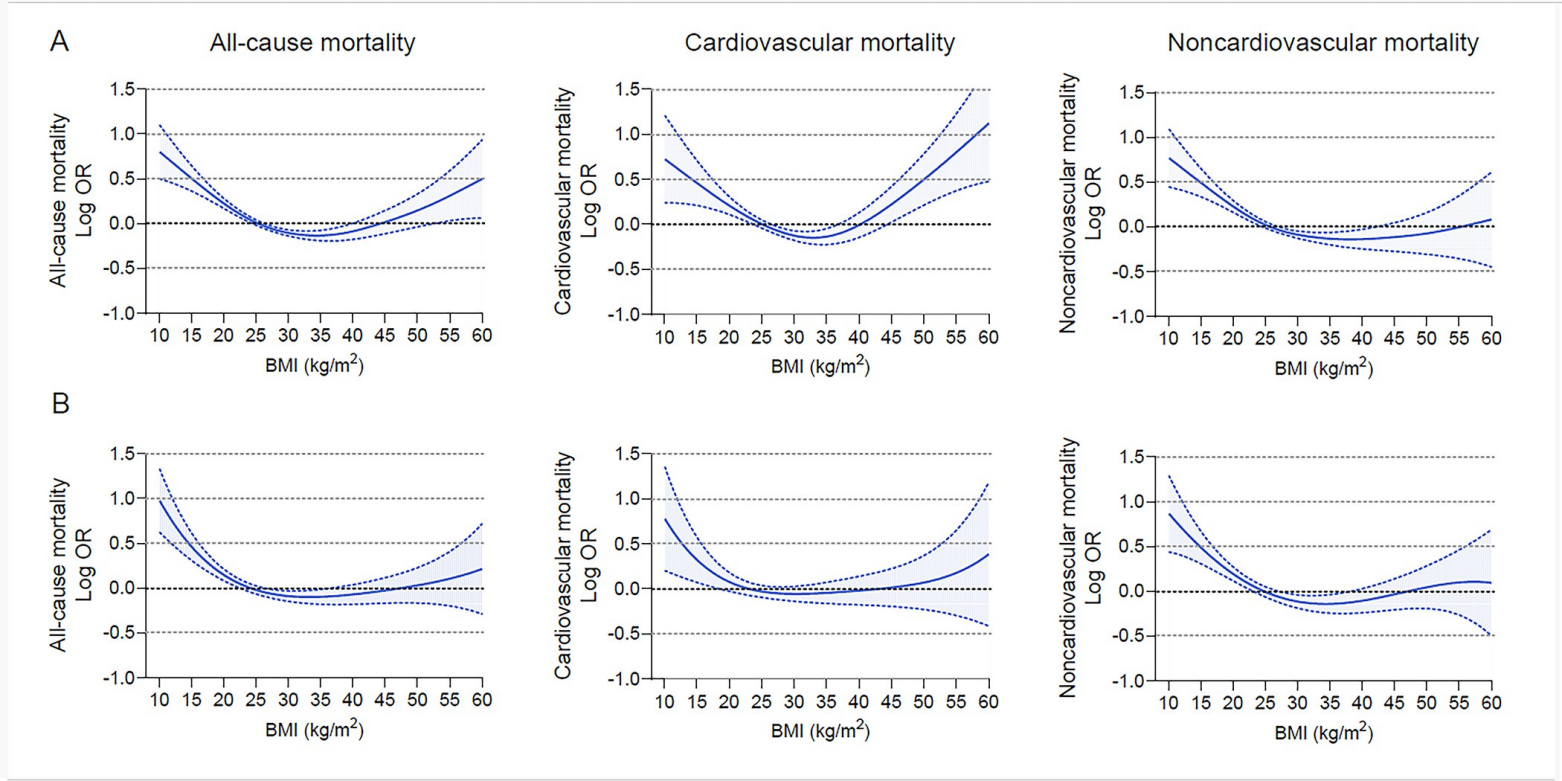
Multivariable adjusted odds ratios for primary outcomes by continuous BMI among men and women. (A) Adjusted odds ratios and 95% confidence intervals for men. (B) adjusted odds ratios and 95% confidence intervals for women. Outcomes were adjusted for all predefined covariates except sex.

**Fig 5:**
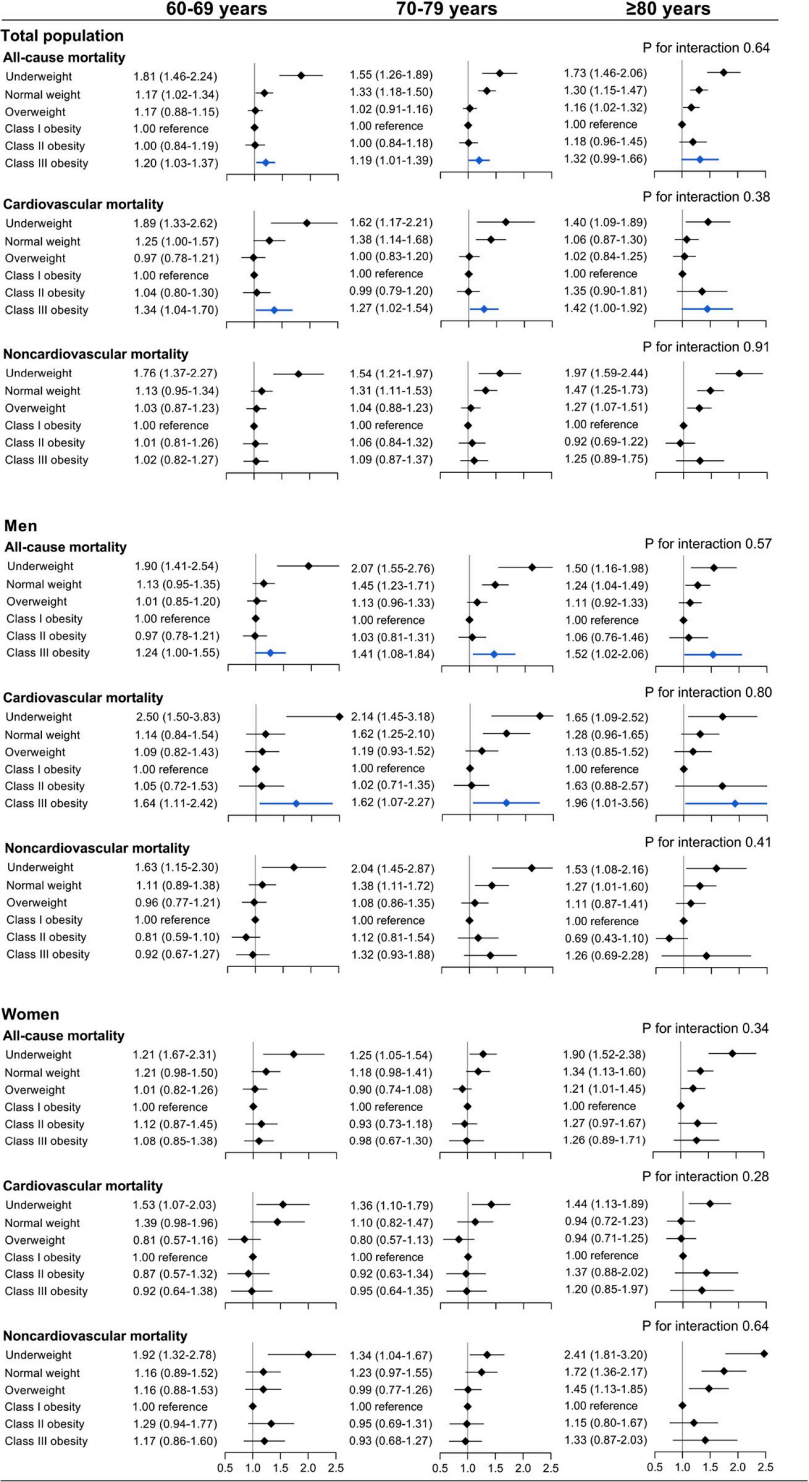
Multivariable adjusted odds ratios for primary outcomes by age, sex and BMI. All-cause mortality, cardiovascular mortality, and noncardiovascular mortality by categorical BMI and ages 60–69, 70–79, and ≥80 years among overall population, men and women. Outcomes were adjusted for all predefined covariates except stratified variables.

We observed a significant effect modification by sex on the association between BMI and cardiovascular death (P for interaction = 0.01). Class III obesity was associated with a greater risk of cardiovascular death among men (OR, 1.68 [95% CI, 1.30–2.18]) but not among women (OR, 0.93 [95% CI, 0.73–1.19]) (Fig 3 and S1 Table). Using adjusted OR curves, there was a U-shaped association between BMI and cardiovascular mortality among men and a reverse J-shaped association among women (Fig 4). No significant sex interactions were found for the association between BMI and the secondary outcomes (S2 and S3 Figs). We further examined the associations between BMI and the primary outcomes by age strata of 60 to 69, 70 to 79, and ≥80, and no significant age interaction was found for all-cause and cause-specific mortality (Fig 5).

### Sensitivity analyses

After excluding patients with early deaths in the first 24 hours of ICU admission, the estimated risk of all-cause and cause-specific mortality did not change much (S4 Fig). When the analysis was restricted to participants without a history of cancer, no evidence was found of substantial alteration in the relationship between BMI and mortality (S5 Fig), nor was any evidence found when the analysis was restricted to those without diabetes (S6 Fig). A complete case analysis by excluding all missing data did not modify the results of the primary analysis (S7 Fig). Cox proportional hazards model analysis also showed consistent results (S8 Fig). Finally, our findings remained robust after additional adjustment for several hospital-level confounding factors (S9 Fig).

## Discussion

In this large multicenter cohort study, we investigated the effect of obesity on ICU outcomes in older critically ill patients and found that: (1) the association between BMI and all-cause and cardiovascular mortality was U-shaped, with an increased risk in severely obese individuals, independent of multisystem critical illness and other traditional risk factors; (2) the risk of cause-specific mortality may not be identical between the sexes, and severe obesity was more strongly associated with cardiovascular mortality in older male patients than their female counterparts; and (3) there was a monotonic positive association between BMI and major composite adverse events including overall mortality, mechanical ventilation, and vasoactive drug usage. These findings suggest that the protective effect of obesity does not appear to apply to short-term ICU outcomes in older critically ill patients, which is mainly because of the significant increase in all-cause and cardiovascular mortality in severely obese individuals, particularly in men, as well as the greater burden of mechanical ventilation associated with obesity.

Previous studies have reported heterogeneous effects of obesity on mortality in older populations. Some studies showed a monotonic inverse association between BMI and mortality [14,15], and others showed a U-shaped association pattern [7,16]. A Danish study further reported that the BMI-mortality association may be age sensitive and is decreasingly U-shaped with advancing age [17]. Most of these studies were conducted in relatively healthy older volunteers or older subjects in primary care settings. Recently, the survival benefit of obesity has been described in critically ill patients, including medical and surgical ICU patients and ICU discharged survivors [18,19]. However, data on older critically ill patients are lacking. In this study, we found a U-shaped association of BMI with all-cause and cardiovascular mortality in critically ill patients over 60 years old. Underweight individuals had the highest risk of death, followed by those with normal weight and class III obesity, whereas those with overweight and class I and II obesity had roughly equivalent low risks. These findings support the protective effect of overweight or mild obesity on survival in older critically ill patients. However, this protective effect cannot be extended to those with severe obesity. Possible mechanisms by which adipose tissue exerts its beneficial role include immune response regulation, inflammatory preconditioning, endotoxin neutralization, higher energy reserve, and sufficient adrenal steroid synthesis [20]. It is worth noting that the structure and function of adipose tissue is dynamically changing and severe obesity still exerts adverse effects on physiological homeostasis, particularly cardiometabolic imbalance, through the expression of detrimental adipokines [20].

In the current analysis, the estimated risk inflection points of all-cause death, cardiovascular death and noncardiovascular death were BMI of 30.9, 31.0 and 30.7 kg/m^2^, respectively, lending further credence to the view that BMI values around the range of overweight or mild obesity may be protective of clinical outcomes among older patients. These cutoffs are slightly higher than the ranges of 26–27 reported in the Canadian Age-Specific BMI Threshold Study [21] and the US NHANES study [22] and the range of 24–25.9 reported in the Chinese Elderly Population Study [23], which may be due to the differences in the included populations. Adipose tissue is a specialized energy-storing organ, which serves as a crucial regulator of systemic energy homeostasis [24]. Given the substantial nutritional consumption and extra importance of nutritional reserves in older critically ill patients, a higher BMI inflection point of mortality risk is plausible.

We found a sex-specific effect of BMI on mortality risk in older critically ill patients. Severe obesity among men increased cardiovascular mortality by 68% compared with their reference counterparts, whereas no such increase was observed among women. In an age-stratified analysis, the association between severe obesity and cardiovascular death remained evident among men, which dominated the association pattern between severe obesity and all-cause mortality. These findings are consistent with the potential biological mechanisms of action of adipose tissue. As an important endocrine and paracrine organ, the adipose tissue exerts a bidirectional and reversible regulatory effect on local biology by secreting a variety of adipocytokines, microparticles and gaseous mediators [25]. The effect of the adipose tissue varies considerably depending on its distribution, quality and local biological properties. Men tend to accumulate more visceral fat and less subcutaneous fat than women [26]. It has been widely confirmed that visceral rather than subcutaneous adipose tissue had a straightforward relevance to cardiovascular risk [27]. Moreover, visceral adipose tissue shows an age-dependent increase, while subcutaneous adipose tissue shows the opposite tendency [28].

A strong, progressive association between BMI category and mechanical ventilation was observed, consistent with the results of two studies on COVID-19 that the dependence on mechanical ventilation increased with higher BMI [29,30]. The underlying mechanism is that obesity increases the ventilatory burden through disadvantageous respiratory mechanics (reducing lung and chest wall compliance and increasing functional residual capacity) and detrimental adipokine expression (reducing anti-inflammatory adiponectin and increasing proinflammatory leptin, resistin and visfatin) [31]. We did not find a direct association between BMI and vasoactive drug usage, consistent with previous reports that obese patients have substantial individual variability in response to haemodynamic support medications, with no consistent dose-response relationship [32,33].

According to these findings, we have some considerations. Because of the extra concerns on nutritional status and muscle mass loss, optimal BMI for the older has been considered to be around the range of overweight or mildly obese, leading to an entrenched and somewhat misleading notion of the obesity paradox, that higher BMI is better and even without considering a clear plateau. Our findings support the protective effect of appropriate adipose reserve, but these results cannot be extended to severely obese individuals. Overweight and mild obesity may be a sign of better nutritional status, which is conducive to getting through the early stages of ICU treatment. However, the markedly increased cardiovascular mortality due to severe obesity in older men remains an obvious obstacle to ICU survival. In addition, obesity also accelerates the decline in respiratory function and leads to greater reliance on mechanical ventilation, indicating excessive medical resource utilization and healthcare economic burden. These findings suggest that severe obesity remains a major challenge for older critically ill patients and that targeted weight management strategies for this population are urgently needed.

### Strengths and limitations

This study was conducted in a multicenter database with a large sample of older critically ill patients, allowing for a more detailed BMI classifications to explore the association between a broader range of BMI and clinical outcomes. We extensively adjusted for potential confounding factors, analyzed the association of BMI with multiple adverse ICU outcomes, and confirmed the robustness of the results in multiple sensitivity analyses. Several limitations should be considered. First, given the inherent limitation of observational studies, we cannot prove a causal relationship between obesity and mortality. Second, residual confounding cannot be completely avoided. For example, smoking is related to lower body weight and increased health risks, but data on smoking status and alcohol consumption are not available in the current database, which may distort regression estimates. Third, we have no information on abdominal obesity or regional body fat distribution, such as waist circumference, waist-height ratio, or dual energy X-ray absorptiometry parameters, which may add valuable information. Finally, there are racial or regional differences in the prevalence and classification of obesity. Therefore, considering that the majority of the current cohort is Caucasian, these results cannot be extrapolated to other ethnic groups.

## Conclusion

The coexistence of ageing and obesity poses a great challenge to the management of critical illness. The findings of this study suggest that the obesity paradox does not appear to apply to short-term ICU outcomes in older critically ill patients, and those with severe obesity have significantly increased all-cause and cardiovascular mortality, particularly in men.

## Supporting information

S1 Checklist STROBE checklist. (DOCX)

S1 Fig Distribution of BMI categories. (DOCX)

S2 Fig Secondary outcomes with categorical BMI by sex. (DOCX)

S3 Fig Secondary outcomes with continuous BMI by sex. (DOCX)

S4 Fig Excluding patients died within 24 hours. (DOCX)

S5 Fig Excluding patients with cancer. (DOCX)

S6 Fig Excluding patients with diabetes. (DOCX)

S7 Fig Complete case analysis. (DOCX)

S8 Fig Cox proportional hazards model. (DOCX)

S9 Fig Additional adjustment of hospital-level factors. (DOCX)

S1 Table Primary outcomes with categorical BMI. (DOCX)

S2 Table Outcomes with BMI below or above inflection point. (DOCX)

S3 Table Secondary outcomes with categorical BMI. (DOCX)

S1 Dataset Data file anonymized. (XLS)
