# Supplementary material for: Impact of obesity on intensive care unit outcomes in older patients with critical illness: A cohort study
Source: PLoS One. 2024 Feb 14;19(2):e0297635. doi: 10.1371/journal.pone.0297635 (PMC10866459; doi:10.1371/journal.pone.0297635)
Supplement: S1 Checklist — (DOCX) [file pone.0297635.s001.docx]

S1 Checklist. STROBE checklist.

|  | Item No. | Recommendation | Page  No. | Relevant text from manuscript |
| --- | --- | --- | --- | --- |
| **Title and abstract** | 1 | (*a*) Indicate the study’s design with a commonly used term in the title or the abstract | 1 | Impact of Obesity on Intensive Care Unit Outcomes in Older Patients with Critical Illness: **A Cohort Study** |
|  |  | (*b*) Provide in the abstract an informative and balanced summary of what was done and what was found | 2 | We examine the association between obesity and 30-day mortality and other adverse outcomes in older patients with critical illness. We found that the obesity paradox appears not apply to short-term intensive care unit (ICU) outcomes in older patients with critical illness, mainly due to increased all-cause and cardiovascular mortality in severely obese patients, particularly in men. |
| Introduction | | | |  |
| Background/rationale | 2 | Explain the scientific background and rationale for the investigation being reported | 3 | The obesity paradox has been described in several clinical conditions, including critical care settings. With the rapid aging of the world's population, ICUs are faced with an increasing need to care for older patients. The impact of obesity on ICU outcomes in older critically ill patients remains unclear. We sought to examine the association between obesity and 30-day mortality and other adverse outcomes in this population. |
| Objectives | 3 | State specific objectives, including any prespecified hypotheses | 3-4 | We sought to determine whether the protective effect of obesity, known as the obesity paradox, remains evident in older critically ill patients. |
| Methods | | | |  |
| Study design | 4 | Present key elements of study design early in the paper | 4-5 | Participants: Older patients (≥ 60 years) in the eICU Database.  Exposure: BMI was stratified as: underweight <18.5 kg/m^2^, normal weight 18.5-24.9 kg/m^2^, overweight 25.0-29.9 kg/m^2^, class I obesity 30.0-34.9 kg/m2, class II obesity 35.0-39.9 kg/m^2^, and class III obesity ≥ 40.0 kg/m^2^.  Outcomes: All-cause mortality and cause-specific mortality within 30 days of ICU admission. |
| Setting | 5 | Describe the setting, locations, and relevant dates, including periods of recruitment, exposure, follow-up, and data collection | 4 | We used the data from the eICU database, which contains electronic medical records from 335 ICUs in 208 U.S. hospitals between 2014 and 2015. We examined 30-day mortality and other adverse outcomes in the ICU. |
| Participants | 6 | (*a*) *Cohort study*—Give the eligibility criteria, and the sources and methods of selection of participants. Describe methods of follow-up  *Case-control study*—Give the eligibility criteria, and the sources and methods of case ascertainment and control selection. Give the rationale for the choice of cases and controls  *Cross-sectional study*—Give the eligibility criteria, and the sources and methods of selection of participants | 4 | The study included older patients (≥ 60 years) in the eICU database. After excluding patients without available weight or height measurements and those with extreme BMIs of less than 10 kg/m^2^ or greater than 60 kg/m^2^, the final analysis included 89234 individuals. We obtained 30-day death information based on the specific time of death after admission to the ICU as recorded in the database. |
|  |  | (*b*) *Cohort study*—For matched studies, give matching criteria and number of exposed and unexposed  *Case-control study*—For matched studies, give matching criteria and the number of controls per case | 7 | \| BMI, kg/m^2^ \| <18.5 \| 18.5-24.9 \| 25.0-29.9 \| 30.0-34.9 \| 35.0-39.9 \| ≥40 \| \| --- \| --- \| --- \| --- \| --- \| --- \| --- \| \| N \| 4016 \| 27286 \| 27616 \| 16543 \| 7632 \| 6141 \| |
| Variables | 7 | Clearly define all outcomes, exposures, predictors, potential confounders, and effect modifiers. Give diagnostic criteria, if applicable | 5 | Exposure: BMI was stratified as: underweight <18.5 kg/m^2^, normal weight 18.5-24.9 kg/m^2^, overweight 25.0-29.9 kg/m^2^, class I obesity 30.0-34.9 kg/m2, class II obesity 35.0-39.9 kg/m^2^, and class III obesity ≥ 40.0 kg/m^2^.  Outcomes: All-cause mortality and cause-specific mortality within 30 days of ICU admission.  Potential confounders: age, sex, ethnicity, mean blood pressure and heart rate, disease severity score (GCS and APACHE score), primary admission disease (circulatory disease, respiratory disease, neurological disease, digestive disease, genitourinary disease, trauma, and other diseases), prior comorbidities (coronary artery disease, stroke/transient ischaemic attacks, diabetes mellitus, hypertension, chronic heart failure, chronic obstructive pulmonary disease, dementia, cirrhosis, peripheral artery disease, renal dysfunction), and important treatments (mechanical ventilation, dialysis, vasoactive drugs). |
| Data sources/ measurement | 8* | For each variable of interest, give sources of data and details of methods of assessment (measurement). Describe comparability of assessment methods if there is more than one group | 4-5 | BMI (kg/m^2^) = weight/height^2^. The weight and height recorded at ICU admission were used for this calculation. BMI was stratified by World Health Organization (WHO) obesity classification. Cause-specific deaths were defined according to the International Classification of Diseases-9 codes. |
| Bias | 9 | Describe any efforts to address potential sources of bias | 6 | We adjusted for a large number of potential confounders. The R package multiple imputation method by chained equations was used to impute missing covariate data. Several sensitivity analyses were performed. |
| Study size | 10 | Explain how the study size was arrived at | 4 | We refer to the previous studies and include a large enough sample to obtain a valid confidence interval. |

Continued on next page

| Quantitative variables | 11 | Explain how quantitative variables were handled in the analyses. If applicable, describe which groupings were chosen and why | 4-5 | BMI was stratified by World Health Organization (WHO) obesity classification, defined as underweight <18.5 kg/m2, normal weight 18.5-24.9 kg/m2, overweight 25.0-29.9 kg/m2, class I obesity 30.0-34.9 kg/m2, class II obesity 35.0-39.9 kg/m2, and class III obesity ≥ 40.0 kg/m2. To facilitate the interpretation of the results, we defined the group with the lowest risk as the reference group, which is class I obesity.  Quantitative variables of covariates are treated as continuous variables unless otherwise specified. |
| --- | --- | --- | --- | --- |
| Statistical methods | 12 | (*a*) Describe all statistical methods, including those used to control for confounding | 6 | Logistic regression model was used to estimate adjusted odds ratios (ORs), and cubic spline curve was used to explore the nonlinear association between BMI and 30-day ICU outcomes. Stratified analysis and sensitivity analysis were also performed.  We used multi-variable adjusted analysis and stratified analysis to control confounding. |
|  |  | (*b*) Describe any methods used to examine subgroups and interactions | 6 | The multiplicative interaction term was tested in the multivariate model. |
|  |  | (*c*) Explain how missing data were addressed | 6 | The R package multiple imputation method by chained equations (N_imputation_= 5) was used to replace the missing covariate data (13.2% for APACHE score, 2.3% for GCS, 1.0% for mean blood pressure, and 0.8% for heart rate). |
|  |  | (*d*) *Cohort study*—If applicable, explain how loss to follow-up was addressed  *Case-control study*—If applicable, explain how matching of cases and controls was addressed  *Cross-sectional study*—If applicable, describe analytical methods taking account of sampling strategy | 6 | We assessed 30-day outcomes using a logistic regression model and no timescale was included. |
|  |  | (*e*) Describe any sensitivity analyses | 6 | (1) An analysis was performed by excluding those who died within 24 hours of ICU admission to mitigate the reverse causality. (2) A model was constructed in patients without a history of cancer to rule out the influence of cancer on the association between BMI and survival. (3) Since diabetes may be an effect mediator of obesity rather than a confounding factor, the analysis excluding individuals with diabetes was performed. (4) A complete case analysis was performed by excluding all missing covariate data to check whether the missing data modified the current findings. (5) A Cox proportional hazards model analysis was performed using ICU length of stay as a potential time scale to account for possible differences in outcomes between statistical methods. (6) Other confounding factors were further addressed, such as admission source, hospital location, ICU bed count, and discharge year, and we constructed an additional model adjusting for these variables in addition to all the other variables. |
| Results | | | | |
| Participants | 13* | (a) Report numbers of individuals at each stage of study—eg numbers potentially eligible, examined for eligibility, confirmed eligible, included in the study, completing follow-up, and analysed | 4 | The study included older patients (≥ 60 years) in the eICU database. After excluding patients without available weight or height measurements and those with extreme BMIs of less than 10 kg/m^2^ or greater than 60 kg/m^2^, the final analysis included 89234 individuals. |
|  |  | (b) Give reasons for non-participation at each stage | 4 | Patients without available weight or height measurements and those with extreme BMIs of less than 10 kg/m^2^ or greater than 60 kg/m^2^ were excluded. |
|  |  | (c) Consider use of a flow diagram |  | We did not use a flowchart due to the simplicity of the included and excluded criteria. |
| Descriptive data | 14* | (a) Give characteristics of study participants (eg demographic, clinical, social) and information on exposures and potential confounders | 8 | See Table 1. |
|  |  | (b) Indicate number of participants with missing data for each variable of interest | 6 | 13.2% for APACHE score, 2.3% for GCS, 1.0% for mean blood pressure, and 0.8% for heart rate |
|  |  | (c) *Cohort study*—Summarise follow-up time (eg, average and total amount) | 5 | 30-day |
| Outcome data | 15* | *Cohort study*—Report numbers of outcome events or summary measures over time | 8 | See Table 1. |
|  |  | *Case-control study—*Report numbers in each exposure category, or summary measures of exposure |  |  |
|  |  | *Cross-sectional study—*Report numbers of outcome events or summary measures |  |  |
| Main results | 16 | (*a*) Give unadjusted estimates and, if applicable, confounder-adjusted estimates and their precision (eg, 95% confidence interval). Make clear which confounders were adjusted for and why they were included |  | Unadjusted and adjusted estimates are shown in tables and figures in the manuscript and supplementary materials. The choice of covariates was based on previous literature and clinical considerations. |
|  |  | (*b*) Report category boundaries when continuous variables were categorized |  | Yes. |
|  |  | (*c*) If relevant, consider translating estimates of relative risk into absolute risk for a meaningful time period |  |  |

Continued on next page

| Other analyses | 17 | Report other analyses done—eg analyses of subgroups and interactions, and sensitivity analyses | 10 | Yes. Sex and age interactions on the association between BMI and outcomes were reported. The results of sensitivity analyses were also reported. |
| --- | --- | --- | --- | --- |
| Discussion | | | | |
| Key results | 18 | Summarise key results with reference to study objectives | 11 | We found that: (1) the association between BMI and all-cause and cardiovascular mortality was U-shaped; (2) the risk of cause-specific mortality may not be identical between the sexes, and severe obesity was more strongly associated with cardiovascular mortality in older male patients than their female counterparts; and (3) there was a monotonic positive association between BMI and major composite adverse events including overall mortality, mechanical ventilation, and vasoactive drug usage. |
| Limitations | 19 | Discuss limitations of the study, taking into account sources of potential bias or imprecision. Discuss both direction and magnitude of any potential bias | 14 | Yes, the limitations are listed. |
| Interpretation | 20 | Give a cautious overall interpretation of results considering objectives, limitations, multiplicity of analyses, results from similar studies, and other relevant evidence | 10-14 | Yes, See discussion section |
| Generalisability | 21 | Discuss the generalisability (external validity) of the study results | 15 | Considering that the majority of the current cohort is Caucasian, these results cannot be extrapolated to other ethnic groups. |
| Other information | |  | | |
| Funding | 22 | Give the source of funding and the role of the funders for the present study and, if applicable, for the original study on which the present article is based |  | No funding. |

*Give information separately for cases and controls in case-control studies and, if applicable, for exposed and unexposed groups in cohort and cross-sectional studies.

**Note:** An Explanation and Elaboration article discusses each checklist item and gives methodological background and published examples of transparent reporting. The STROBE checklist is best used in conjunction with this article (freely available on the Web sites of PLoS Medicine at http://www.plosmedicine.org/, Annals of Internal Medicine at http://www.annals.org/, and Epidemiology at http://www.epidem.com/). Information on the STROBE Initiative is available at www.strobe-statement.org.
