# Supplementary figures and images for: Impact of obesity on intensive care unit outcomes in older patients with critical illness: A cohort study
Source: PLoS One. 2024 Feb 14;19(2):e0297635. doi: 10.1371/journal.pone.0297635 (PMC10866459; doi:10.1371/journal.pone.0297635)

**S1 Figure.** Distribution of BMI categories


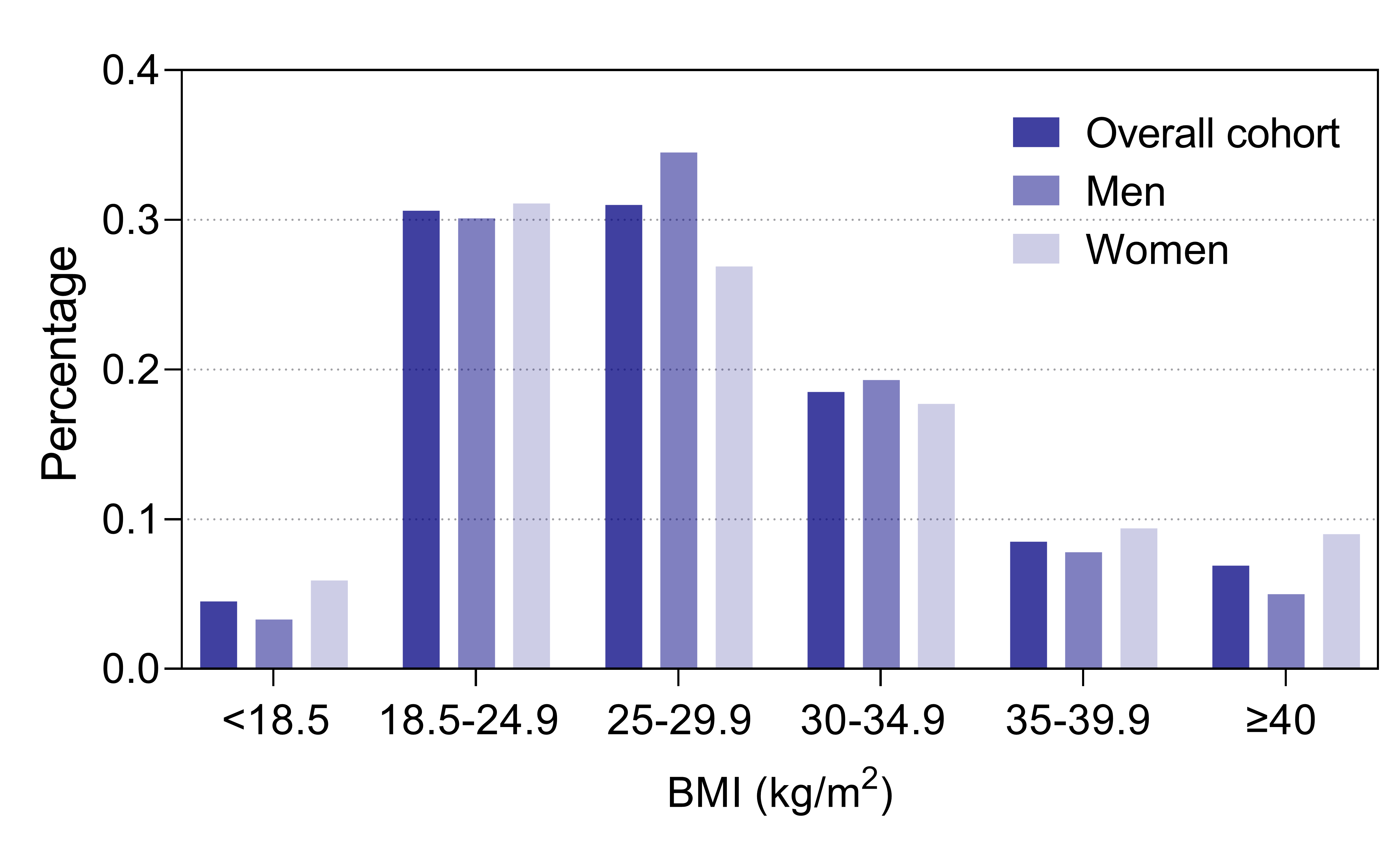

Supplement: S1 Fig — (DOCX) [file pone.0297635.s002.docx]
