# Supplementary material for: Impact of obesity on intensive care unit outcomes in older patients with critical illness: A cohort study
Source: PLoS One. 2024 Feb 14;19(2):e0297635. doi: 10.1371/journal.pone.0297635 (PMC10866459; doi:10.1371/journal.pone.0297635)
Supplement: S4 Fig — (DOCX) [file pone.0297635.s005.docx]

**S4 Figure.** Multivariable adjusted odds ratios for primary outcomes by categorical BMI after excluding patients died within 24 hours of admission (n=87066)


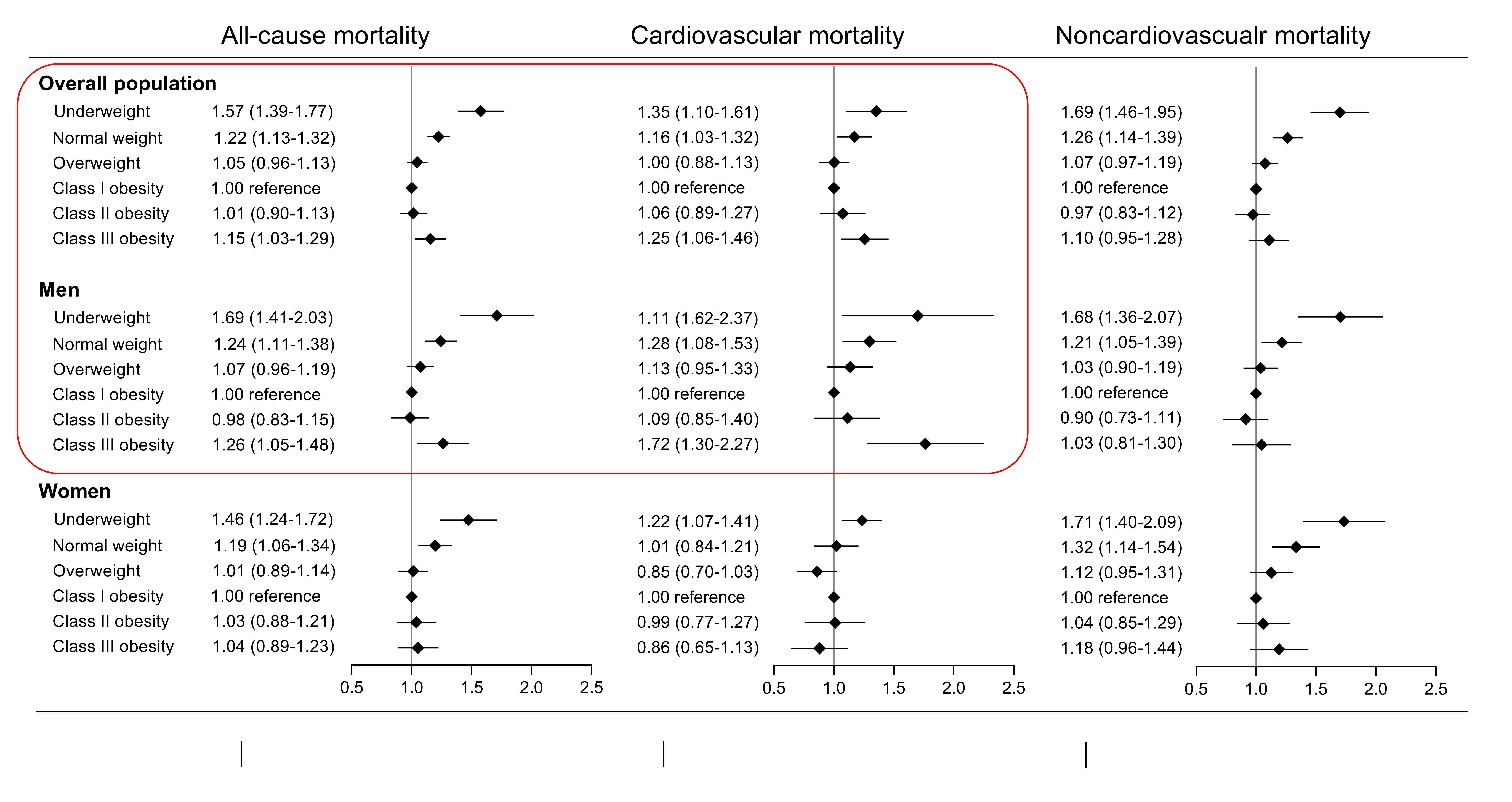


Outcomes were adjusted for age, sex, ethnicity, mean blood pressure, heart rate, GCS, APACHE score, primary admission disease (circulatory disease, respiratory disease, neurological disease, digestive disease, genitourinary disease, trauma, and other diseases), prior comorbidities (coronary artery disease, stroke/transient ischemic attacks, diabetes mellitus, hypertension, chronic heart failure, chronic obstructive pulmonary disease, dementia, cirrhosis, peripheral artery disease, renal dysfunction), mechanical ventilation, dialysis, vasoactive drugs.
