# Supplementary material for: Impact of obesity on intensive care unit outcomes in older patients with critical illness: A cohort study
Source: PLoS One. 2024 Feb 14;19(2):e0297635. doi: 10.1371/journal.pone.0297635 (PMC10866459; doi:10.1371/journal.pone.0297635)
Supplement: S3 Table — (DOCX) [file pone.0297635.s013.docx]

**S3 Table.** Univariable and multivariable adjusted odds ratios for secondary outcomes by categorical BMI

| BMI category | Underweight  18.5 kg/m^2^ | normal weight  18.5-24.9 kg/m^2^ | Overweight  25-29.9 kg/m^2^ | class I obesity  30-34.9 kg/m^2^ | class II obesity  35-39.9 kg/m^2^ | class III obesity  ≥40 kg/m^2^ |
| --- | --- | --- | --- | --- | --- | --- |
|  | OR (95% CI) | OR (95% CI) | OR (95% CI) | Reference | Reference | OR (95% CI) |
| **Major adverse events** | |  |  |  |  |  |
| **Overall population** | |  |  |  |  |  |
| Model I | 1.25 (1.17, 1.35) | 1.00 (0.96, 1.04) | 0.94 (0.90, 0.98) | 1 | 1.16 (1.10, 1.23) | 1.45 (1.36, 1.54) |
| Model II | 1.25 (1.16, 1.34) | 0.99 (0.95, 1.03) | 0.93 (0.90, 0.97) | 1 | 1.17 (1.10, 1.23) | 1.46 (1.38, 1.56) |
| Model III | 0.90 (0.83, 0.97) | 0.89 (0.84, 0.93) | 0.92 (0.88, 0.97) | 1 | 1.12 (1.04, 1.20) | 1.33 (1.24, 1.43) |
| **Men** |  |  |  |  |  |  |
| Model I | 1.34 (1.20, 1.49) | 0.99 (0.93, 1.05) | 0.91 (0.86, 0.97) | 1 | 1.18 (1.09, 1.28) | 1.53 (1.40, 1.68) |
| Model II | 1.31 (1.17, 1.47) | 0.97 (0.91, 1.02) | 0.90 (0.85, 0.95) | 1 | 1.19 (1.10, 1.29) | 1.56 (1.42, 1.71) |
| Model III | 0.89 (0.78, 1.02) | 0.86 (0.80, 0.92) | 0.91 (0.85, 0.97) | 1 | 1.16 (1.05, 1.27) | 1.40 (1.26, 1.57) |
| **Women** |  |  |  |  |  |  |
| Model I | 1.21 (1.10, 1.33) | 1.01 (0.95, 1.07) | 0.98 (0.92, 1.04) | 1 | 1.14 (1.05, 1.24) | 1.41 (1.30, 1.53) |
| Model II | 1.22 (1.11, 1.34) | 1.01 (0.95, 1.08) | 0.98 (0.92, 1.04) | 1 | 1.14 (1.05, 1.24) | 1.40 (1.29, 1.52) |
| Model III | 0.92 (0.82, 1.03) | 0.93 (0.86, 1.00) | 0.94 (0.87, 1.01) | 1 | 1.08 (0.98, 1.19) | 1.29 (1.17, 1.42) |
| **Mechanical ventilation** | |  |  |  |  |  |
| **Overall population** | |  |  |  |  |  |
| Model I | 0.99 (0.92, 1.08) | 0.88 (0.84, 0.92) | 0.89 (0.85, 0.94) | 1 | 1.20 (1.13, 1.27) | 1.54 (1.44, 1.64) |
| Model II | 1.02 (0.94, 1.10) | 0.90 (0.86, 0.95) | 0.91 (0.87, 0.95) | 1 | 1.19 (1.11, 1.26) | 1.50 (1.41, 1.60) |
| Model III | 0.70 (0.63, 0.77) | 0.80 (0.76, 0.85) | 0.88 (0.84, 0.93) | 1 | 1.15 (1.07, 1.23) | 1.40 (1.30, 1.51) |
| **Men** |  |  |  |  |  |  |
| Model I | 1.05 (0.92, 1.18) | 0.87 (0.82, 0.92) | 0.89 (0.84, 0.94) | 1 | 1.25 (1.15, 1.37) | 1.61 (1.47, 1.78) |
| Model II | 1.04 (0.92, 1.18) | 0.88 (0.83, 0.94) | 0.90 (0.84, 0.95) | 1 | 1.25 (1.15, 1.36) | 1.59 (1.44, 1.75) |
| Model III | 0.68 (0.59, 0.79) | 0.78 (0.72, 0.84) | 0.90 (0.84, 0.97) | 1 | 1.24 (1.12, 1.37) | 1.48 (1.32, 1.66) |
| **Women** |  |  |  |  |  |  |
| Model I | 0.96 (0.87, 1.07) | 0.90 (0.84, 0.96) | 0.90 (0.84, 0.97) | 1 | 1.15 (1.05, 1.25) | 1.49 (1.37, 1.63) |
| Model II | 1.00 (0.90, 1.12) | 0.93 (0.87, 1.00) | 0.92 (0.86, 0.98) | 1 | 1.13 (1.03, 1.23) | 1.44 (1.32, 1.57) |
| Model III | 0.71 (0.62, 0.80) | 0.83 (0.77, 0.90) | 0.86 (0.80, 0.94) | 1 | 1.06 (0.96, 1.18) | 1.35 (1.22, 1.50) |
| **Vasoactive drug usage** | |  |  |  |  |  |
| **Overall population** | |  |  |  |  |  |
| Model I | 1.07 (0.90, 1.27) | 1.03 (0.93, 1.14) | 1.10 (0.99, 1.21) | 1 | 1.07 (0.93, 1.22) | 0.98 (0.84, 1.13) |
| Model II | 1.13 (0.95, 1.34) | 1.05 (0.95, 1.15) | 1.09 (0.99, 1.20) | 1 | 1.08 (0.95, 1.24) | 1.01 (0.86, 1.17) |
| Model III | 0.99 (0.83, 1.18) | 1.00 (0.90, 1.11) | 1.10 (0.99, 1.21) | 1 | 1.02 (0.88, 1.17) | 0.89 (0.76, 1.04) |
| **Men** |  |  |  |  |  |  |
| Model I | 0.99 (0.76, 1.28) | 0.97 (0.85, 1.10) | 1.03 (0.91, 1.16) | 1 | 0.98 (0.81, 1.17) | 0.99 (0.80, 1.23) |
| Model II | 1.01 (0.78, 1.31) | 0.96 (0.84, 1.09) | 1.02 (0.90, 1.15) | 1 | 0.99 (0.82, 1.19) | 1.01 (0.81, 1.25) |
| Model III | 0.85 (0.65, 1.12) | 0.91 (0.79, 1.04) | 1.03 (0.90, 1.17) | 1 | 0.93 (0.77, 1.13) | 0.85 (0.68, 1.07) |
| **Women** |  |  |  |  |  |  |
| Model I | 1.22 (0.97, 1.54) | 1.14 (0.98, 1.32) | 1.19 (1.02, 1.38) | 1 | 1.22 (1.00, 1.49) | 1.04 (0.84, 1.28) |
| Model II | 1.30 (1.03, 1.64) | 1.18 (1.01, 1.37) | 1.19 (1.02, 1.39) | 1 | 1.21 (0.99, 1.47) | 1.03 (0.83, 1.27) |
| Model III | 1.19 (0.93, 1.52) | 1.15 (0.98, 1.35) | 1.21 (1.03, 1.42) | 1 | 1.14 (0.92, 1.40) | 0.95 (0.76, 1.19) |

Model I, unadjusted. Model II, adjusted for age, sex and ethnicity. Model III, adjusted for age, sex, ethnicity, mean blood pressure, heart rate, GCS, APACHE score, primary admission disease (circulatory disease, respiratory disease, neurological disease, digestive disease, genitourinary disease, trauma, and other diseases), prior comorbidities (coronary artery disease, stroke/transient ischemic attacks, diabetes mellitus, hypertension, chronic heart failure, chronic obstructive pulmonary disease, dementia, cirrhosis, peripheral artery disease, renal dysfunction), dialysis.
